# Effects of wood resource size and decomposition on hyphal outgrowth of a cord-forming basidiomycete, *Phanerochaete velutina*

**DOI:** 10.1038/s41598-020-79058-8

**Published:** 2020-12-14

**Authors:** Yu Fukasawa, Koji Kaga

**Affiliations:** Graduate School of Agricultural Science, Tohoku University, 232-3 Yomogida, Naruko, Osaki, Miyagi 989-6711 Japan

**Keywords:** Microbiology, Fungi, Fungal ecology

## Abstract

To assess the relationship between resource use and hyphal growth in a cord-forming basidiomycete, *Phanerochaete velutina*, soil microcosm experiments were conducted using wood blocks of three different sizes in three different soil quantities, thereby simulating the different amounts of available nutrients. The highest percentage weight loss was observed in the smallest wood blocks after a 27-d incubation period in soil microcosms, although the percentage weight loss over the 2-month pure culture colonization prior to inoculation was not significantly different among various block sizes. The greatest hyphal outgrowth was also observed in the smallest wood blocks and was positively associated with wood decay. The slopes of the regression lines between hyphal coverage and percentage wood mass loss were identical among different wood sizes, but the slopes between hyphal coverage and absolute wood mass loss were steeper in the smaller wood blocks than that in largest one. These results suggest that the level of intensity of mycelial foraging for new resources in the soil depends on the percentage of the amount of wood resource utilized, and not on the absolute amount of carbon obtained from the wood.

## Introduction

Fungi are one of the primary decomposers of dead plant tissues in forest ecosystems. Cord-forming basidiomycetes are particularly important due to their persistent linear organs that consist of several aggregated hyphae (cords) that connect multiple dead plant tissues, such as leaves, branches, and trunks, and forms large networks in the forest floor that efficiently translocate carbon and nutrients^1^. These fungi are abundant on the forest floor^2^, often occupying large areas and being long-lived^3^ to dynamically allocate their biomass to forage for new resources^4^. The translocation of nutrients along the mycelial cords results in highly dynamic soil environments in which nutrients rarely remain concentrated at the point of first introduction^5^. A better understanding of resource use and developmental cues in mycelial cords is essential to understand carbon and nutrient cycling and redistribution on the forest floor.

**Figure 1:**
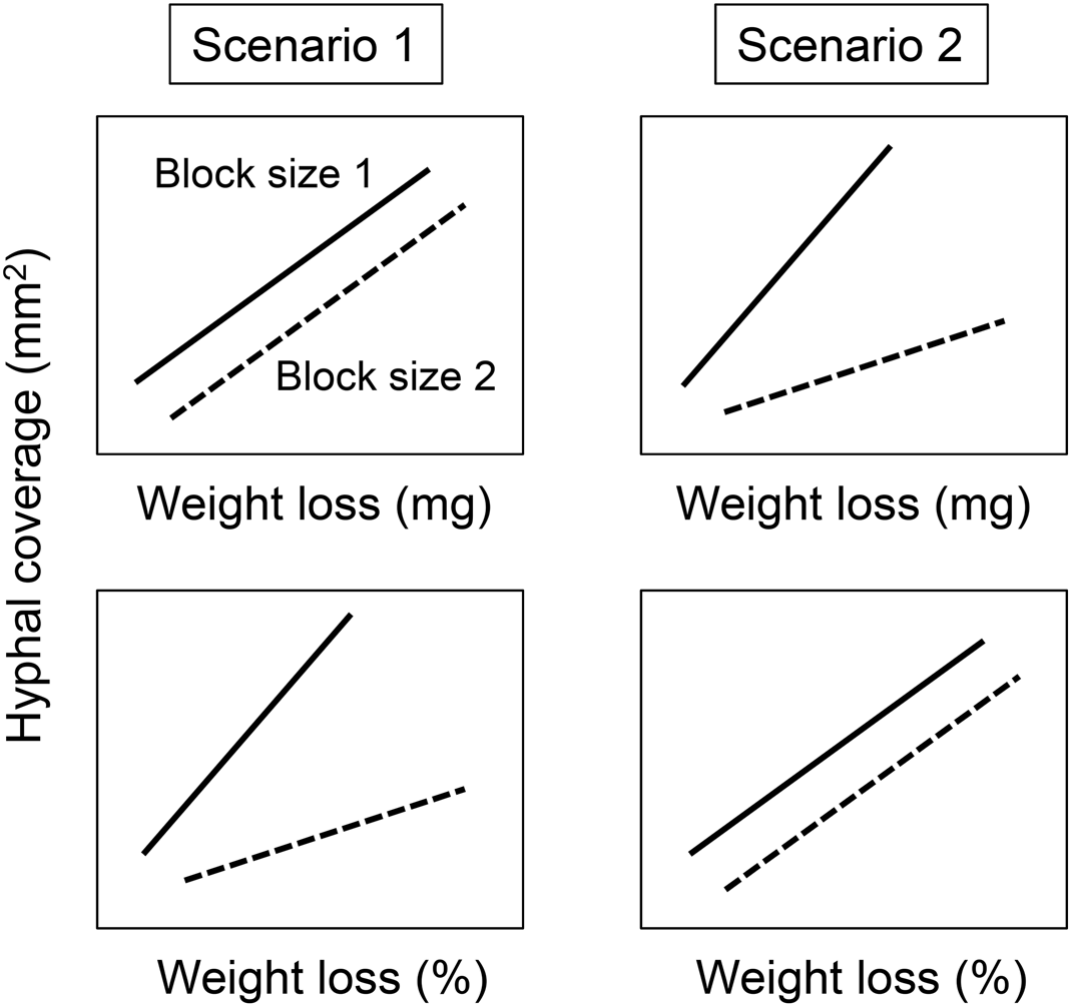
Scheme of the two hypothetical scenarios tested in the present study.

The foraging behavior of cord-forming fungi is a well-explored topic^2,4,6^. Using a soil microcosm system, previous studies revealed that the development of hyphal network outgrowths into the soil from wood inoculum is associated with the amount of wood resource and wood decomposition^7,8^. Nutrient and carbon transport along the network is also associated with the size and quality of wood resources^9–12^. These results may indicate that the growth of mycelial cord network is driven by simple source-sink relationships between resources and hyphae, similar to the hypothesis of tip-directed bulk flow that was suggested for fine-scale individual hyphae^13^. However, developing models to explain the dynamics of the whole mycelial network system remains challenging as they show self-organization, adaptive networks, and dynamic behavior^14,15^. Mycelial outgrowths from the inoculum into the soil to search for new resources do correlate with the decay of the wood inoculum^7^. Moreover, the foraging mycelia of a cord-forming fungus, *Phanerochaete velutina*, can recognize the size of newly found wood resource and determine whether to stay in the original inoculum or move to the new wood resource^16^. Nevertheless, it remains unclear whether the growth of mycelium into the soil depends on the obtained carbon from inoculum through decomposition or whether it grows into the soil because the remaining resource level is depleted. This question can be addressed by checking the relationships between hyphal growth and absolute or percentage wood mass loss in different wood sizes (Fig. 1). If the regression slope between hyphal growth and absolute wood mass loss is not significantly different among different wood inoculum sizes (Scenario 1), the fungal outgrowth may respond to the amount of carbon obtained from the inoculum. However, if the slope is significantly different among inoculum wood sizes and the slope between hyphal growth and percentage wood mass loss among the inoculum wood sizes is not (Scenario 2), the fungus may respond to the percentage of the resources utilized in the inoculum for foraging outgrowth. It means that the fungus can recognize the whole wood mass it occupies and the remaining resource levels.

In the present study, we compared the relationship between hyphal growth and absolute or percentage wood mass loss in different wood block sizes by preparing 50 soil microcosm replicates for each size. We used *P. velutina* as a model, because it is a cosmopolitan fungus and a well-studied species in the field of mycelial network behavioral research^2,4,6,16^. Since the geometry of the solid resource is essential for microbial decomposition^17^, we prepared three types of wood blocks with ranges of surface area and soil contact to unit volume (Table 1). We also tested three different soil amounts because the energy allocation strategies of fungal mycelia may vary with the system’s nutrient availability, as the fungus maintains the carbon and nutrient balance in its hyphal body^18^.

**Table 1:** Geometry of the wood blocks in three different sizes used in the present study.

	0.5 cm^3^	1 cm^3^	2 cm^3^
Surface area (cm^2^)	4	6	10
Surface area: volume	8	6	5
Soil contact (cm^2^)	1	1	2
Soil contact: volume	2	1	1

## Results

**Figure 2:**
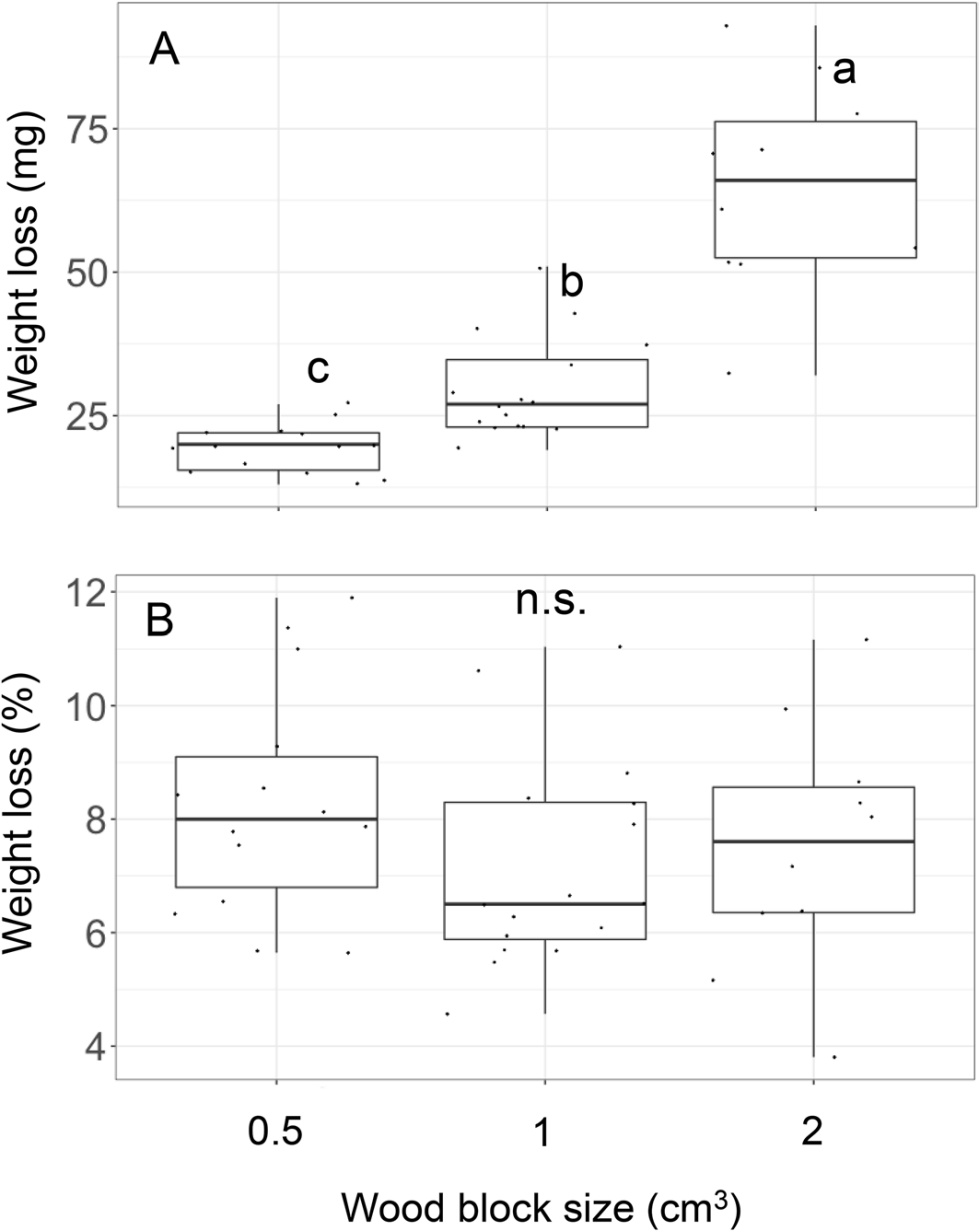
Absolute weight loss (**A**) and percentage weight loss relative to the original weight (**B**) of wood blocks after 2 months of *Phanerochaete velutina* colonization. Different lowercase letters indicate significant differences between wood block sizes (Nemenyi test, *P* < 0.05). N.s. indicates no significant difference between wood blocks (Kruskal–Wallis test, chi-squared = 2.2151, df = 2, *P* = 0.3304). *N* = 14 for 0.5 cm^3^, 16 for 1 cm^3^, and 10 for 2 cm^3^ wood blocks.

The absolute weight loss of the pine wood blocks during the 2-month colonization of *P. velutina* in pure culture varied among wood blocks of different sizes (Fig. 2A). The largest wood blocks (2 cm^3^) showed the greatest weight loss (median = 66 mg), followed by 1 cm^3^ wood blocks (27 mg), and 0.5 cm^3^ wood blocks (20 mg) (Nemenyi test, *P* < 0.05). However, the weight loss percentage relative to the original weight of wood blocks was not significantly different between wood blocks of various sizes (median = 7.7%) (Fig. 2B).

**Figure 3:**
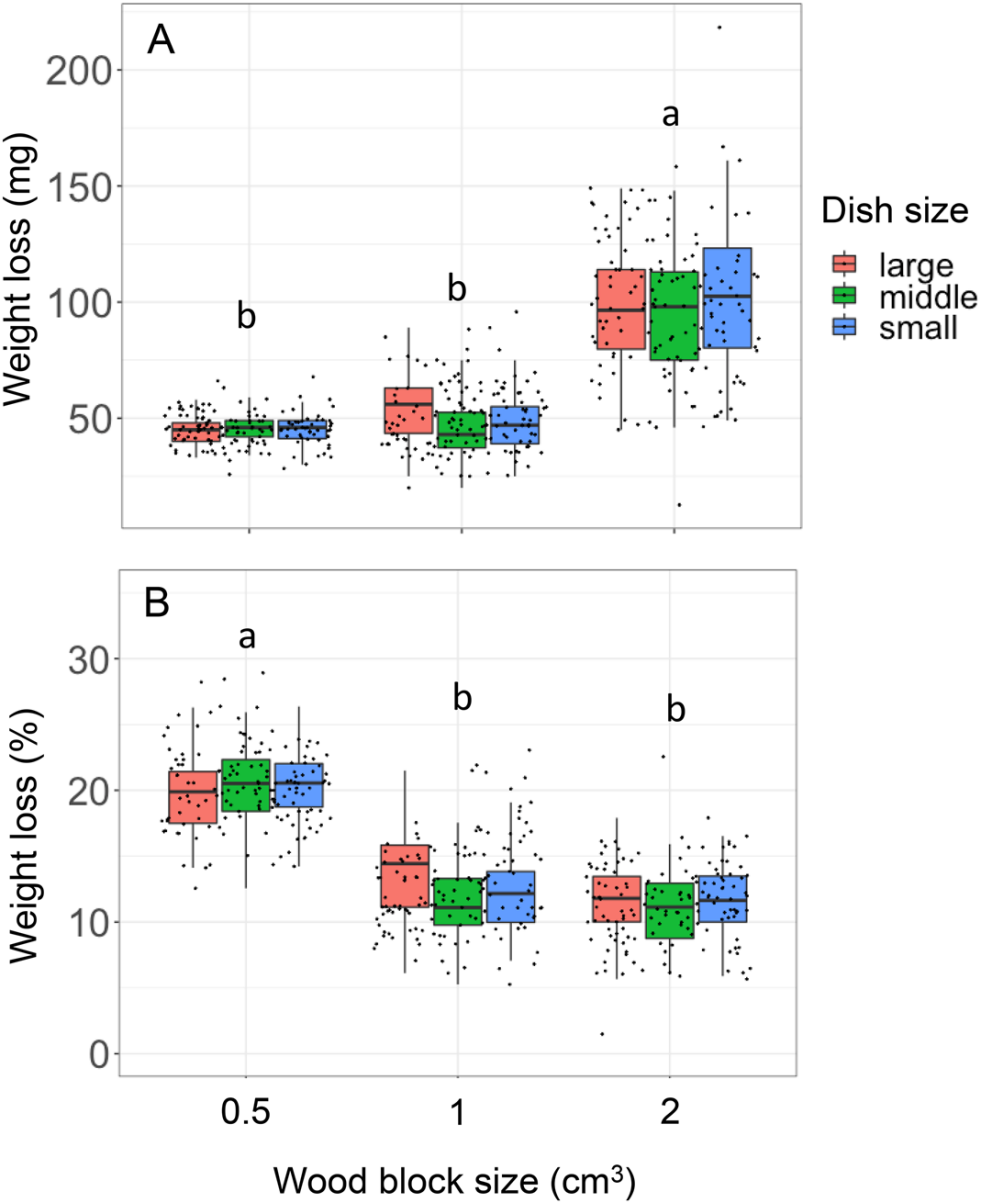
Absolute weight loss (**A**) and percentage weight loss relative to the original weight (**B**) of wood blocks after 2 months of *P. velutina* colonization + 27 days of soil microcosm incubation. Different lowercase letters indicate significant differences between wood block sizes (Nemenyi test, *P* < 0.05). *N* = 50.

After microcosm incubation, the absolute weight loss of the 2 cm^3^ wood blocks (median = 99 mg) during the whole incubation period (2-month fungal colonization + 27 days in soil microcosm) was greater than that of 1 cm^3^ wood blocks (48 mg) and 0.5 cm^3^ wood blocks (46 mg) (Nemenyi test, *P* < 0.05) (Fig. 3A). However, the weight loss percentage relative to the original weight of the wood blocks was greater in the smallest wood blocks (median = 20.2%) than in 1 cm^3^ (12.3%) and 2 cm^3^ (11.7%) wood blocks (Fig. 3B). Soil dish size and its interaction with wood block size did not have significant effects on either the absolute or percentage weight loss (Table 2).

**Table 2:** Results of linear regression showing the estimated coefficients for the weight loss of wood blocks and hyphal coverage growing on the soil.

	Weight loss		Hyphal coverage^†^
	Absolute	Percent^†^	
Inoculum size	37.96***	–0.33***	–2.92***
Dish size	NS	NS	SIG
Inoculum*Dish	NS	NS	SIG
Absolute weight loss	–	–	0.05***

NS, not significant; SIG, significant.

***P < 0.001.

**Figure 4:**
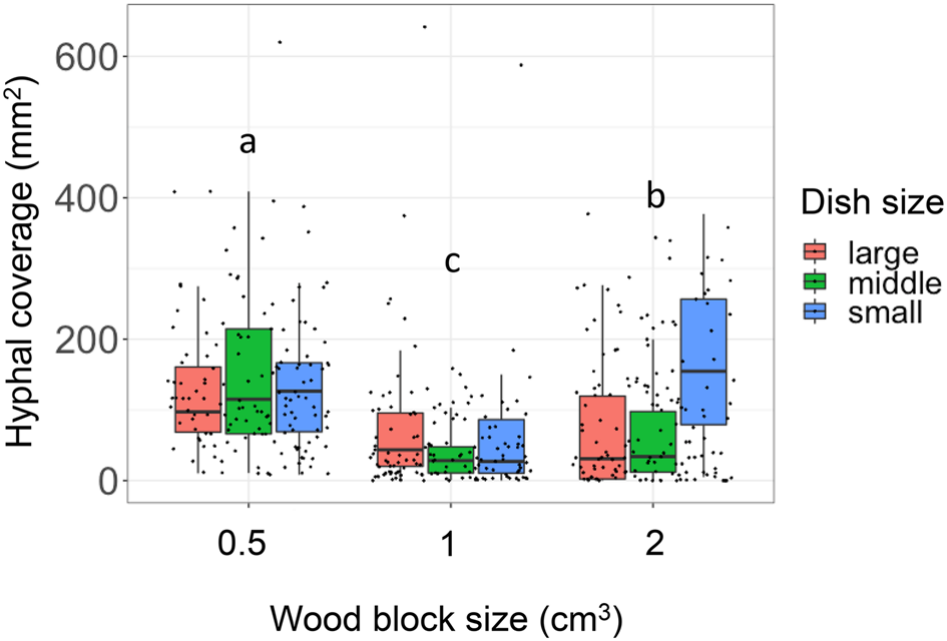
Hyphal coverage (mm^2^) on unsterilized soil after 27 days of incubation. Different lowercase letters indicate significant differences between wood block sizes (Nemenyi test, *P* < 0.05). *N* = 50.

**Figure 5:**
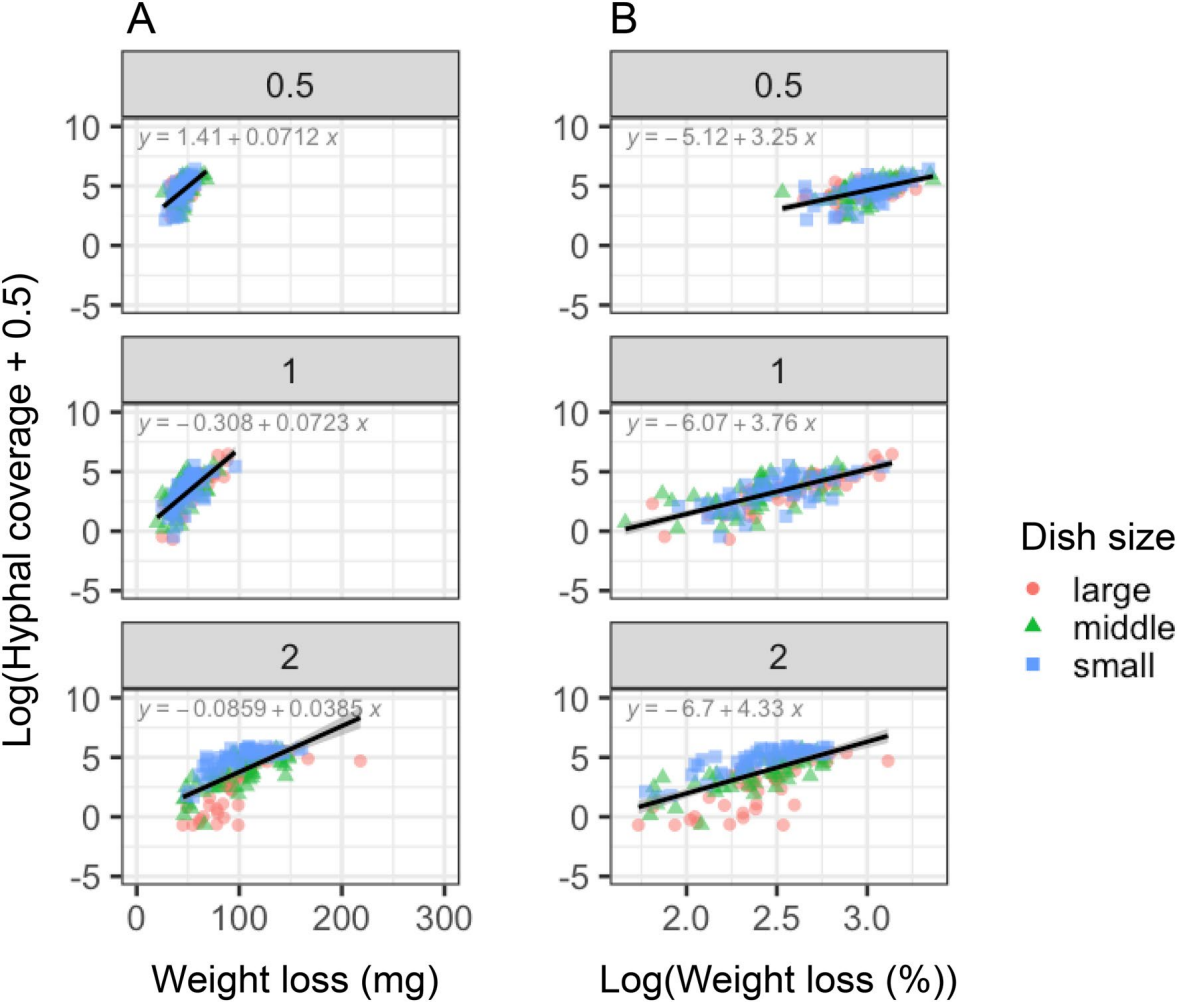
Relationships between wood weight loss and hyphal coverage (mm^2^) growing from 0.5 cm^3^, 1 cm^3^, and 2 cm^3^ wood blocks onto the soil surface after 27 days of incubation. (**A**) Absolute weight loss (mg) and (**B**) percentage weight loss relative to the original weight of the wood blocks. Black line shows regression line with 95% confidential interval for pooled data from all dish sizes. *N* = 50.

After microcosm incubation, the coverage of hyphae growing from the wood blocks into the soil was greatest in 0.5 cm^3^ wood blocks (median = 113.59 mm^2^), followed by 2 cm^3^ wood blocks (70.9 mm^2^), and 1 cm^3^ wood blocks (32.3 mm^2^) (Fig. 4). Hyphal coverage was positively associated with wood weight loss (Table 2; Fig. 5). Soil dish size and the interaction between wood block size and dish size had significant effects on hyphal coverage (Table 2). The regression slopes between absolute weight loss and hyphal coverage were significantly different among various wood block sizes, particularly the 2 cm^3^ wood blocks (Fig. 5; Table 3), whereas the slopes between percentage weight loss and hyphal coverage were not significantly different among various wood block sizes (Fig. 5, Table 3).

**Table 3:** Results of ANCOVA showing the *F*-value and probability levels (Bonferroni corrected) of interaction between weight loss and hyphal coverage (log-transformed) for the three wood block sizes.

	0.5 cm^3^	1 cm^3^	2 cm^3^
0.5 cm^3^	–	0.938	2.6734
1 cm^3^	0.0126	–	1.3711
2 cm^3^	6.806*	21.477***	–

The three values at the bottom left side of the table show the *F*-values of the relationship between absolute weight loss and hyphal coverage. The three values at the upper right side of the table show the *F*-values of the relationship between percentage weight loss (log-transformed) and hyphal coverage (log-transformed).

*P < 0.05; ***P < 0.001.

The soil moisture content after the experiment was significantly different across dish sizes; the median values for the large, middle, and small dishes were 37.01%, 35.38%, and 33.27%, respectively (Kruskal–Wallis test, chi-squared = 281.13, df = 2, *P* = 2.2e^–16^, *N* = 450).

## Discussion

The mycelia in the present study occupied the entire volume of wood blocks and decayed the wood at the same rate in pure culture regardless of wood block size. Therefore, while absolute wood weight loss depended on the volume of wood block, the percentage weight loss did not, as shown in Fig. 2. In contrast, the percentage weight loss of the smallest wood block (0.5 cm^3^) was significantly greater than that of the larger wood blocks after the soil microcosm experiment (Fig. 3). Although the soil microcosm incubation period (27 days) was less than half of the pure *P. velutina* culture period (2 months), the percentage weight loss of the 0.5 cm^3^ wood blocks after the soil microcosm was twice as more than that after the pure culture period, indicating that wood decay was stimulated to a greater degree in 0.5 cm^3^ wood blocks than in 1 cm^3^ and 2 cm^3^ wood blocks in soil microcosm. A higher decay rate on smaller substrates is a well-known phenomenon reported from both field observation^17,19^ and laboratory experiments^11,12,20–22^. The physical efficiency of CO_2_ efflux from smaller substrates due to their large surface to volume ratio may not be the reason for this difference in decay rate because we did not find any difference in percentage weight loss between wood blocks of different sizes in pure culture (Fig. 2); a significant difference in percentage weight loss was recorded only after soil microcosm incubation (Fig. 3). Oberle et al.^17^ revealed in their field experiments that the relative area of soil contact to the unit wood volume is a critical determinant of wood decay rate. They discussed that the movement of soil nutrients and water into the wood, and the colonization of decomposer microbes, which merit the microbial decomposition of wood and certainly a function of the area of soil contact, is a driver of this effect. In the present study, the area of soil contact to unit wood volume is 2, 1, and 1 for 0.5 cm^3^, 1 cm^3^, and 2 cm^3^ wood blocks, respectively (Table 1). This difference is consistent with the percentage weight loss data, which was doubled in 0.5 cm^3^ blocks compared to that of 1 cm^3^ and 2 cm^3^ blocks (Fig. 3). However, the movement of nutrients, water, and microbes associated with decay might not be the reason of the difference in decay rate observed in the present study, because, there was no difference in the decay rate of various block sizes in pure culture, and all the wood blocks were already colonized by the decomposer fungi.

An alternative explanation for the observed higher decay rate on smaller substrates would be associated with the biotic interactions among microbes. Although the overall conditions are markedly different between pure cultures on nutrient agar plates and cultures in soil microcosm, a particularly notable difference is that the latter system is not sterile and includes a variety of soil microorganisms. Therefore, two mechanisms could explain why smaller wood blocks decay faster than larger ones. First, energy loss due to competition with other microorganisms in soil may be compensated for by a stimulated decay rate^23–25^. Since 0.5 cm^3^ wood blocks have doubled soil contact per unit volume (Table 1), the mycelia colonizing 0.5 cm^3^ blocks have double energy costs per unit volume due to the need to protect their territory than mycelia colonizing larger wood blocks. Second, some soil fungi “steal” low-molecular weight carbohydrates (such as mono- and disaccharides) produced from the wood decay activities of structural decomposers, and promote decomposition by reducing the negative feedback effects of the low-molecular weight carbohydrates on fungal enzyme production^26^. This effect may be greater in wood blocks that have larger soil contact because soil microbes can “steal” the decay products of wood-inhabiting basidiomycetes from larger areas. In any case, we assumed that *P. velutina* was responsible for the decomposition of wood structural components in the present study. Although we did not check the viability of *P. velutina* mycelia in the wood blocks by re-isolating the fungi from harvested wood blocks, our observations certainly suggest that the mycelia were active and occupied the entire volume of the wood blocks as the hyphae grows from the wood radially and covers the surfaces of the blocks during the incubation period*.*

The smallest wood blocks showed the largest mycelial growth on the surrounding soil surface (Fig. 4). This result was apparently inconsistent with previous studies that reported that mycelium growing out from smaller wood blocks showed smaller hyphal coverage or biomass^7,16^. The area of soil contact usually becomes larger in wood blocks with larger volumes, which merit hyphal outgrowth. However, our results showed that the hyphal coverage of 2 cm^3^ blocks are rather smaller than that of 0.5 cm^3^ wood blocks (Fig. 4). These results indicated that the difference of soil contact area might not be the main driver of hyphal outgrowth onto the soil. If the weight loss of wood is considered as a covariant, our results showed that the regression slope between hyphal coverage and percent wood weight loss was identical among different wood sizes regardless of the difference in weight loss (Fig. 5), whereas the regression slope between hyphal coverage and absolute wood weight loss was steeper in smaller wood. These results suggest that the intensity level of mycelial foraging for new resources in the soil depends on the percentage of wood resource utilized, and not on the amount of carbon obtained from wood. It was already known that the mycelia of *P. velutina* can recognize resource quantity and manage their foraging behavior accordingly^2,16^. However, we report that the mycelia of *P. velutina* may recognize the whole mass of the resource it occupied and use the information of remaining resource quantity to regulate hyphal outgrowth intensity, as revealed by the experiments with controlled geometry of wood resources and regression analyses of numerous replicates.

Although we applied linear regression on the relationship between weight loss of wood blocks and hyphal coverage because it is relatively in the early stage of wood decomposition, such relationship between resource utilization and hyphal outgrowth may not be linear in the later stages of decay, and hyphal outgrowth from well-decayed wood may be reduced due to energy starvation. Bolton and Boddy^7^ reported that hyphal outgrowth was significantly greater in 0.5 cm^3^ inoculum pre-colonized for 56 d compared with 154 d inoculum, whereas hyphal outgrowth from 8 cm^3^ inoculum pre-colonized for 154 d was significantly larger than that of 56 d inoculum. Therefore, more studies are needed to elucidate fungal cognition and behavioral responses in relation to the resource quantity and quality. For example, experiments with a unified area of soil contact and larger range of wood volume would be reasonable. In addition, exploring the mechanisms by which mycelia recognize the quality or quantity of resources will undoubtedly be of immense value in future studies, and this may require molecular analytical techniques, such as bioimaging and transcriptomic analyses^27,28^.

Although the soil was carefully sprayed every week, the soil moisture content after the experiment was significantly different across the dish sizes. Therefore, it is not clear whether the reason for the significant effect of dish size on hyphal coverage detected in the present study was moisture content or nutrient amount. Although we did not measure the actual water potential of the soil after the experiment, it was initially prepared to be – 0.012 MPa. Donnelly and Boddy^29^ reported that small difference in water potential around this level does not change the hyphal outgrowth of *P. velutina* greatly. Since the hyphal coverage was substantially less than the whole soil area, differences in soil amount (i.e. nutrient amount) may not have limited hyphal growth.

## Methods

### Fungal culture and wood block preparation

Kiln-dried pine (*Pinus densiflora*) wood was cut into blocks of three sizes [0.5 × 1 × 1 cm (0.5 cm^3^), 1 × 1 × 1 cm (1 cm^3^), or 2 × 1 × 1 cm (2 cm^3^)] and dried at 70 °C to constant weight. These blocks have different surface area and soil contact area to unit volume (Table 1). Weighed blocks were numbered, soaked overnight in DH_2_O, and then autoclaved at 121 °C for 20 min in double, sealed autoclave bags. Autoclaving was repeated three times with 1 day intervals. Sterilized wood blocks were placed onto cultures of *P. velutina* (NITE Biological Resource Center, NBRC culture collection, #110,184, originally isolated from *P. densiflora* deadwood by YF) grown on 0.5% malt extract agar (5 g malt extract, 15 g agar; Nakalai Tesque, Kyoto, Japan) in non-vented Petri dishes (2 cm thick, 9 cm in diameter). The plates were sealed with Parafilm® (Bemis Company Inc., Oshkosh, USA) and incubated in the dark at 20 °C for 2 months before use in the soil microcosm experiment. In total, we prepared 490 inoculated blocks (164 blocks of 0.5 cm^3^, 166 blocks of 1 cm^3^, and 160 blocks of 2 cm^3^). We harvested 14 blocks of 0.5 cm^3^, 16 blocks of 1 cm^3^, and 10 blocks of 2 cm^3^ to measure weight loss after 2 months of incubation (i.e. after *P. velutina* colonization but before microcosm incubation). The harvested blocks were dried at 70 °C to constant weight and weighed. We defined absolute weight loss and percentage weight loss as follows:

$$\begin{aligned} {\text{Absolute weight loss}} & = {\text{DW}}_{{{\text{t}}1}} {-}{\text{DW}}_{{{\text{t}}0}} \\ {\text{Percentage weight loss}} & = \left( {{\text{DW}}_{{{\text{t}}1}} {-}{\text{DW}}_{{{\text{t}}0}} } \right)/{\text{DW}}_{{{\text{t}}0}} \\ \end{aligned}$$

where DW_t0_ is the dry weight of the block before it was incubated with the fungus; DW_t1_ is the dry weight of the block after the treatment. The remaining 450 blocks (150 blocks for each block size) were used in the microcosm experiment.

### Microcosm preparation

The soil was collected from the top 10 cm (A layer) in a deciduous mixed forest dominated by *Quercus serrata* and *Pinus densiflora* in Miyagi, Japan (38°37′N, 140°48′E, 129 m.a.s.l.). After sieving on site (10 mm mesh), the soil was air-dried, sieved again through a 2 mm mesh, and frozen at – 30 °C over 48 h to kill soil invertebrates. The soil was then rehydrated with DH_2_O (300 ml per 1 kg dried soil), transferred to plastic dishes of three different sizes, and smoothed and compacted to about 5 mm depth. The weight of rehydrated soil transferred to each dish was 200 g for large dishes (576 cm^2^), 60 g for middle-sized dishes (152 cm^2^), and 20 g for small dishes (64 cm^2^). One wood block, from which surface mycelia and excess agar had been removed using a razor blade, was placed at the center of each dish. Fifty replicates were performed for each experiment (3 wood block sizes × 3 dish sizes = 9 experiments, total of 450 blocks).

### Microcosm incubation

Each dish was weighed, and the lost water was replaced every week by spraying DH_2_O evenly across the soil surface until each dish reached its original mass. Dishes were covered with a lid, stacked, and sealed in polythene bags to reduce water loss, and were incubated at 20 °C in the dark for 27 days. The dishes were randomly repositioned every week during incubation to avoid the possible effects of location within the incubator on hyphal growth. Dishes were photographed at the end of the incubation period using a Canon EOS Kiss camera mounted on a stand at a height of 46 cm. Then, the wood blocks were harvested, dried at 70 °C, and weighed. After the incubation, the absolute and percentage weight loss was calculated for each wood block as described above. After harvesting, the soil moisture content was calculated as the percentage of water weight relative to wet soil weight for each dish.

### Image analysis

Images were analyzed using ImageJ (National Institute of Health, USA). A 2 cm calibration line was drawn electronically using a ruler next to each dish. The edge of each soil dish and wood block were removed by windowing, and the resulting images were converted to black and white with a manually set threshold. The mycelia and soil were indicated by black and white pixels, respectively, allowing hyphal coverage (mm^2^) to be determined.

### Statistical analysis

All statistical analyses were conducted in R 3.6.1^30^. The absolute and percentage weight loss of the blocks and hyphal coverage on the soil were compared across wood block sizes using the Nemenyi test (PMCMR package) with Bonferroni correction of the probability values. The soil moisture content after the experiment was also compared across dish sizes using the Nemenyi test with Bonferroni correction of the probability values. The effects of wood block size, dish size, and interaction between these two factors on the absolute and percentage weight loss of wood blocks were evaluated using a linear model (LM_1) by lm function in R. Furthermore, the effects of wood block size, dish size, interactions between these two factors, and absolute weight loss on hyphal coverage were evaluated using LM_2. The percentage weight loss and hyphal coverage were log-transformed before analyses. Due to very small data values, we used an offset of + 0.5 for the log-transformation to satisfy the assumptions of normality^31^. The percentage weight loss of the wood blocks was not used as a factor in LM_2 because it was highly correlated with wood block size. The level of collinearity between predictor variables was checked by calculating the variance inflation factor (VIF, car backage); all VIF values were < 3, indicating low levels of multicollinearity in the models. Analysis of covariance (ANCOVA) was performed to compare the relationship between hyphal coverage and weight loss (absolute or percentage) among inoculum wood sizes; hyphal coverage (log-transformed) was the response variable, absolute loss and percentage loss (log-transformed) were the covariates, and block size was the factor. It was performed with the lm function in R.

## Data Availability

The datasets generated during the current study are available from the corresponding author on reasonable request.
